# Inhibition of HIV-1 entry by extracts derived from traditional Chinese medicinal herbal plants

**DOI:** 10.1186/1472-6882-9-29

**Published:** 2009-08-05

**Authors:** In-Woo Park, Changri Han, Xiaoping Song, Linden A Green, Ting Wang, Ying Liu, Changchun Cen, Xinming Song, Biao Yang, Guangying Chen, Johnny J He

**Affiliations:** 1 Department of Microbiology and Immunology, Indiana University School of Medicine, Indianapolis, IN 46202, USA; 2 Center for AIDS Research, Indiana University School of Medicine, Indianapolis, IN 46202, USA; 3 Department of Chemistry, Hainan Normal University, Haikou 571158, PR China

## Abstract

**Background:**

Highly active anti-retroviral therapy (HAART) is the current HIV/AIDS treatment modality. Despite the fact that HAART is very effective in suppressing HIV-1 replication and reducing the mortality of HIV/AIDS patients, it has become increasingly clear that HAART does not offer an ultimate cure to HIV/AIDS. The high cost of the HAART regimen has impeded its delivery to over 90% of the HIV/AIDS population in the world. This reality has urgently called for the need to develop inexpensive alternative anti-HIV/AIDS therapy. This need has further manifested by recent clinical trial failures in anti-HIV-1 vaccines and microbicides. In the current study, we characterized a panel of extracts of traditional Chinese medicinal herbal plants for their activities against HIV-1 replication.

**Methods:**

Crude and fractionated extracts were prepared from various parts of nine traditional Chinese medicinal herbal plants in Hainan Island, China. These extracts were first screened for their anti-HIV activity and cytotoxicity in human CD4+ Jurkat cells. Then, a single-round pseudotyped HIV-luciferase reporter virus system (HIV-Luc) was used to identify potential anti-HIV mechanisms of these extracts.

**Results:**

Two extracts, one from *Euphorbiaceae*, *Trigonostema xyphophylloides *(TXE) and one from *Dipterocarpaceae*, *Vatica astrotricha *(VAD) inhibited HIV-1 replication and syncytia formation in CD4+ Jurkat cells, and had little adverse effects on host cell proliferation and survival. TXE and VAD did not show any direct inhibitory effects on the HIV-1 RT enzymatic activity. Treatment of these two extracts during the infection significantly blocked infection of the reporter virus. However, pre-treatment of the reporter virus with the extracts and treatment of the extracts post-infection had little effects on the infectivity or gene expression of the reporter virus.

**Conclusion:**

These results demonstrate that TXE and VAD inhibit HIV-1 replication likely by blocking HIV-1 interaction with target cells, i.e., the interaction between gp120 and CD4/CCR5 or gp120 and CD4/CXCR4 and point to the potential of developing these two extracts to be HIV-1 entry inhibitors.

## Background

Human immunodeficiency virus type 1 (HIV-1) causes acquired immune deficiency syndrome (AIDS) [1,2]. CD4+ T lymphocytes are the natural target of HIV-1 infection [3]. At the cellular level, HIV-1 life cycle begins with binding of HIV-1 gp120 to cellular receptors CD4 and chemokine receptors CCR5 or CXCR4 that are expressed on the surface of HIV-1 target cells, followed by gp41 conformational change, which in turn leads to virus-cell membrane fusion and entry of the viral core (nucleocapsid) into the cytoplasm [4-6]. The virion core undergoes uncoating, the viral RNA genome is converted into proviral DNA by the virally encoded enzyme reverse transcriptase (RT) [7]. The DNA enters the nucleus and is covalently integrated into the genome of the host cell by the second virally encoded enzyme integrase (IN) [8-10]. The integrated viral DNA serves as the template for viral transcription and synthesis of various components of progeny viruses [7]. Progeny viruses are assembled on and budded through the plasma [11,12]. As a result, the progeny viruses become encapsulated by a layer of membrane that also harbors the viral envelope glycoproteins [6]. Concomitant with budding, a third virally encoded enzyme protease (PR) processes the core proteins into their final forms, and the virion undergoes a morphologic change known as maturation [7,13]. This final step primes the progeny viruses for the next round of infection.

In parallel with these progresses made in our understanding of basic HIV-1 virology and pathogenesis is development of anti-HIV-1 therapeutics. The primary targets for anti-HIV-1 therapeutic development have been two virally encoded enzymes: RT and PR. The Food and Drug Administration (FDA) has approved a total of 21 anti-HIV-1 drugs, a majority of these drugs are HIV-1 RT and PR inhibitors. Various combinations of these inhibitors, so-called highly active anti-retroviral therapy (HAART) is very effective in suppressing viral replication and has led to a significant reduction in the mortality rate of the disease, increase in the lifespan of HIV/AIDS patients and improvement of the quality of life of these patients [14-16]. However, issues such as viral reservoirs, drug resistance, high dosages and frequencies, and high cost, have led to a significant crisis in the management of HIV/AIDS patients, particularly in developing nations, where there is the greatest need [17-19]. It has become evident that HAART does not offer a complete solution to the problem. Meanwhile, relatively fewer anti-HIV-1 therapeutics have been developed to target other steps of HIV-1 life cycle including entry, fusion, and integration. On the other hand, recent trials on anti-HIV-1 vaccines and microbicides have shown that some of current vaccine and microbicide strategies not only did not prevent but actually increased HIV-1 infection and transmission risks [20-23]. Therefore, additional and alternative anti-HIV-1 therapeutic strategies are desperately needed to be explored and developed to fight this virus from destroying the immune system of infected individuals and from spreading the virus to others.

In the current study, we investigated a panel of traditional Chinese medicinal herbal extracts obtained from plants in Hainan Island, China, a geographically unique tropical/subtropical region for their activities against HIV-1 replication. We demonstrate that extracts from *Euphorbiaceae*, *Trigonostema xyphophylloides *(TXE) and *Dipterocarpaceae*, *Vatica astrotricha *(VAD) both block HIV-1 replication at the entry step. These results point to the potential of developing these plant extracts as anti-HIV-1 entry inhibitors.

## Methods

### Preparation of plant extracts

All plants used in this study were collected at national tropical forest parks in Hainan island, the People's Republic of China (P.R. China) including Jianfengling, Bawangling, or Mt. Diaoluo (Table 1). Scientific names and classification of these plants were validated by Prof. Qiongxin Zhong, a plant taxonomist at Department of Biology Hainan Normal University, Haikou, P. R. China. Samples of these plants were kept at the Hainan Provincial Key Laboratory of Tropical Pharmaceutical Herbal Chemistry, Haikou, P. R. China. Plant samples were first air dried, grinded and continued to be dried in a pressurized oven at 40°C and 0.08 MPa. The dried and grinded materials were then subjected to 3 rounds of refluxing extraction in 75% ethanol at 80°C. The ethanol extracts were then concentrated to become ointment in a revolving depressurized vacuum evaporator at 55°C. The ointment was further lyophilized to the final form of powder and stored at a desiccator. The powders were dissolved in dimethyl sulfoxide (DMSO) at a concentration of 100 mg/ml with gentle shaking overnight at room temperature. Then, the mixtures were removed of any remaining undissolved substances by centrifugation at 3,000 rpm for 10 min followed by filtration through a 0.4 μm syringe filter. The cleared ones were used in all experiments.

**Table 1:** Tropical plants selected to make extracts for anti-HIV screening

*Plant (family, species)*	Sampling location	Sampling parts	Medicinal usage
*Euphorbiaceae, Antidesma ghaesembilla*	Mt. Diaoluo	Stem	Vascuodilation
*Euphorbiaceae, Alchoornea rugosa*	Jianfengling	Stem	Rash
*Euphorbiaceae, Alchoornea rugosa*	Jianfengling	Leaves	Rash
*Euphorbiaceae, Trigonostema xyphophylloides*	Jianfengling	Stem	Asthma
*Euphorbiaceae, Trigonostema xyphophylloides*	Jianfengling	Leaves	Asthma
*Euphorbiaceae, Mallotus furetianus*	Mt. Diaoluo	Stem	Fever
*Euphorbiaceae, Mallotus furetianus*	Mt. Diaoluo	Leaves	Fever
*Euphorbiaceae, Sapium insigne Benth. Et Hook*	Bawangling	Leaves	Snake bite
*Clavicipitaceae, Cordyceps inensis Sacc*	Haikou city	Root	TB, cough, sweating
*Rubiaceae, Saprosma hainanense Merr*	Bawangling	Stem	Bactericidal
*Guttiferae, Calophyllum membranaceum*	Bawangling	Stem	Arthritis, pain
*Dipterocarpaceae, Vatica astrotricha*	Bawangling	Leaves	Bactericidal, hepatitis
*Dipterocarpaceae, Vatica astrotricha*	Bawangling	Stem	Bactericidal, hepatitis

### Cells, HIV-1 HXBc2 viruses and chemicals

Jurkat cells were purchased from American Tissue Culture Collection (ATCC, Manassas, VA) and cultured in RPMI 1640 medium supplemented with 10% fetal bovine serum, 100 units/ml penicillin, and 100 μg/ml streptomycin sulfate. U87.CD4.CXCR4 and U87.CD4.CCR5 cells expressing CD4/CXCR4 and CD4/CCR5 respectively were obtained from the NIH AIDS Reagent Program [24] and cultured in DMEM medium supplemented with 10% fetal bovine serum, 100 units/ml penicillin, and 100 μg/ml streptomycin sulfate. Proviral DNA of T-tropic HIV-1 strain HXBc2 was transfected into Jurkat cells to generate viral inoculums by the DEAE-Dextran method. HIV-1 HXBc2 expresses HIV-1 Eli strain *nef *and all other HXB2 genes. Unless stated otherwise, all chemicals were from Sigma (St. Louis, MO).

### HIV-1 replication assay

One million Jurkat cells in 1 ml culture medium were infected with HIV-1 corresponding to a 10,000 cpm RT activity. At twenty-four hr post-infection, cells were treated with plant extracts at indicated concentrations or equivalent concentrations of the DMSO solvent. Fresh extracts as well as DMSO were added every other day. Meanwhile, the culture supernatants were collected for the RT activity assay. Briefly, 1 ml of the culture supernatant was collected and cleared to remove any cells and cell debris by centrifugation at 1,000 g for 5 min, followed by filtration of the cleared supernatants through a 0.2 μm syringe filter. Virions in the supernatant were pelleted by centrifugation at 12,000 g for 1 hr and the RT activity was determined as described [25,26].

### Cytotoxicity and syncytia formation

The cytotoxicity of the plant extracts was determined using the trypan blue exclusion method. Briefly, Jurkat cells that were exposed to plant extracts in the presence or absence of HIV-1 infection for various lengths of time were stained in 0.2% trypan blue dye and then counted for viable cells under a light microscope. HIV-1-infected Jurkat cells were scored for syncytia formation from 4 random fields from each of the triplicate samples over the course of HIV-1 infection by a light microscope.

### Preparation and infection of HIV-1 pseudotyped viruses

HIV-1 viruses pseudotyped with different envelope proteins were prepared as previously described [27,28]. Briefly, 293T cells (2 × 10^6 ^cells per 10-cm plate) were transfected with 20 μg of HIV-Luc plasmid and 4 μg of pHXB2-env, p89.6-env, pVSV-G, or pcDNA3 by the calcium phosphate precipitation method. Cell culture supernatants were collected 48 hr after medium change, filtered, and saved as virus stocks. For infection, pseudotyped viruses corresponding to a 2,000 cpm RT activity were used to infect target cells. Following 2 hr infection, the cells were removed of remaining viruses by multiple washes with fresh medium. The cells were continued to incubate for 48 hr and then harvested for the Luc activity assay as described [27,28].

### Fractionation of crude extracts of TXE and VAD

TXE and VAD extracts obtained above were suspended in 1.5 L H_2_O and partitioned successively with petroleum ether (PE) (4 × 1.5 L), chloroform (CF) (5 × 1.5 L), ethyl acetate (EA) (5 × 1.5 L), and n-butanol (BT) (5 × 1.5 L) to obtain respective subfractions. The excessive solvents were removed from these subfractions under a reduced pressure to generate ointments. The ointments were lyophilized to the powder form. These subfractions were dissolved in DMSO at a concentration of 100 mg/ml by overnight shaking on a shaker, and the undissolved materials were removed by low speed centrifugation followed by filtration through syringe filter, as described above.

### Data analysis

All values expressed as mean ± SEM, or representative of at least three independent experiments. Comparisons among groups were made using two-tailed Student's *t*-test. A *p *value of < 0.05 was considered statistically significant (*), and *p *< 0.01 highly significant (**).

## Results

### Anti-HIV-1 activity of extracts of traditional Chinese medicinal herbal plants

**Figure 1:**
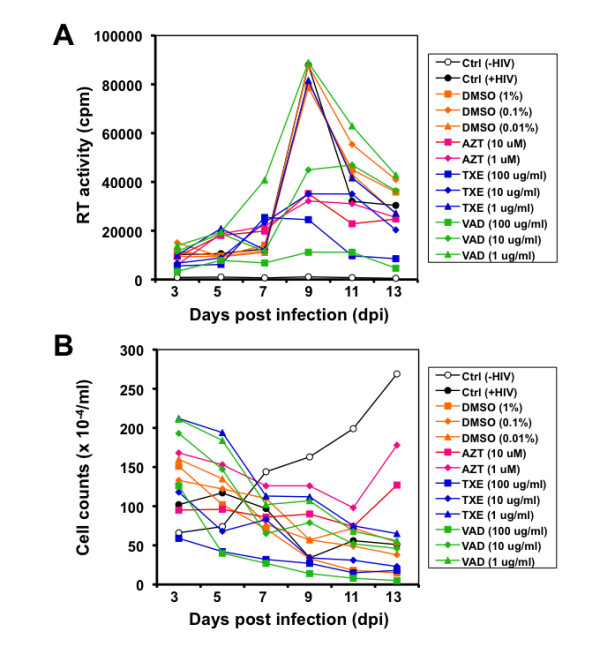
**Effects of the extracts from TXE and VAD on HIV replication and cell survival**. Jurkat cells were infected with HIV-1 HXB2 and then exposed to the extracts 24 hr post infection. Fresh extracts were added every other day. Meanwhile, culture supernatants were collected for the RT activity assay (**A**), and aliquots of cells were stained with trypan blue dye and counted for viable cells (**B**). DMSO was the solvent of the extracts and included as a negative control, while AZT was included as a positive control. In addition, Jurkat cells without HIV infection [Ctrl, (-HIV)] and Jurkat cells with HIV infection but without any treatments [Ctrl, (+HIV)] were also included. These data were representative of three independent experiments.

Traditional Chinese Medicine (TCM) dates back to 2000 to 3000 years. Medicinal herbs are a major component of TCM. It is estimated that over 600 different herbs have been used to treat various human diseases including those caused by virus infection [29]. Hainan Island, the second largest island off the coast of China, is located in the South China Sea and in the tropics at about 18°N latitude. There are about 4,200 plant species, 630 of which are listed as endemic to the island and some are nearly extinct. In this study, we selected 13 medicinal herbal plant parts that have been used to treat various human diseases by local ethnic Chinese in Hainan Island, China (Table 1), extracted these plants with ethanol, and tested them for the anti-HIV activity. We infected CD4+ T lymphocytes Jurkat with a replication-competent T-tropic HIV-1 strain HXB2 and then monitored HIV-1 replication over a course of 2 weeks in the presence of the plant extracts at concentrations of 1, 10, 100 μg/ml. We included the solvent DMSO of the extracts and an HIV-1 RT inhibitor AZT in these experiments. We repeated this initial testing three times. Compared to the untreated control or DMSO treatment, only two of these 13 extracts: one from the stem of *Euphorbiaceae*, *Trigonostema xyphophylloides *(TXE) and the other from the stem of *Dipterocarpaceae*, *Vatica astrotricha *(VAD), displayed significant inhibition of HIV-1 replication at 10 μg/ml or higher at day 9 post infection (dpi) (Figure 1A). As expected, the treatment control AZT inhibited HIV-1 replication. These two extracts at 1 μg/ml and all other extracts showed little effects on HIV-1 replication but dose-dependent anti-HIV activity at 10 μg/ml or higher. We also monitored cell survival of all treatments throughout the experiments by trypan blue dye staining. Compared to the untreated control, both TXE and VAD treatments showed cell growth kinetics similarly to that of DMSO and no treatment controls (Figure 1B). The decline in the number of viable cells in HIV-1-infected cells likely results from the infection-induced cell death, as the cell number began to recover from AZT treatment toward the end of the treatment. These results provide initial evidence that TXE and VAD are inhibitory to HIV-1 replication.

### Cytotoxicity of the extracts

**Figure 2:**
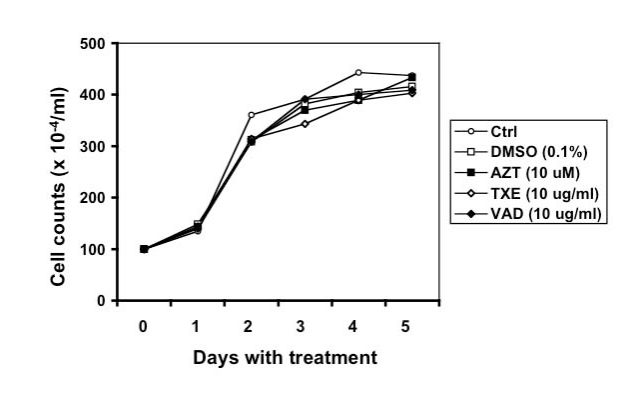
**Effects of the extracts on cell proliferation and survival**. Jurkat cells were exposed to the extracts for various lengths of time as indicated. Fresh extracts were added every other day. Cells without any treatments, treated with DMSO, or AZT were included as controls. Viable cells were determined using the trypan blue dye staining. These data were representative of three independent experiments.

To ascertain and establish non-toxic working concentrations of the extracts, we further determined effects of these extracts on cell survival and growth kinetics in the absence of HIV-1 infection. We treated Jurkat cells with TXE or VAD at 1, 10 and 100 μg/ml and added the fresh extract every other day and monitored cell survival and growth. We included DMSO and AZT as controls in these experiments. Jurkat cells without any treatment were also included as a control. The cell survival and growth kinetics appeared to be indistinguishable among the cells receiving no treatment, AZT (10 μM), and 10 μg/ml TXE and VAD and its corresponding DMSO concentration, i.e., 0.1% (Figure 2). Similar results were obtained between TXE and VAD at 1 and 100 μg/ml and their respective solvent DMSO concentrations 1% and 0.01% (data not shown), suggesting that TXE and VAD are not toxic at a concentration up to 100 μg/ml. Based on these results, we chose the concentration of 10 μg/ml for TXE and VAD extracts for all following mechanistic studies.

### Inhibition of HIV-1-induced syncytia formation by the extracts

**Figure 3:**
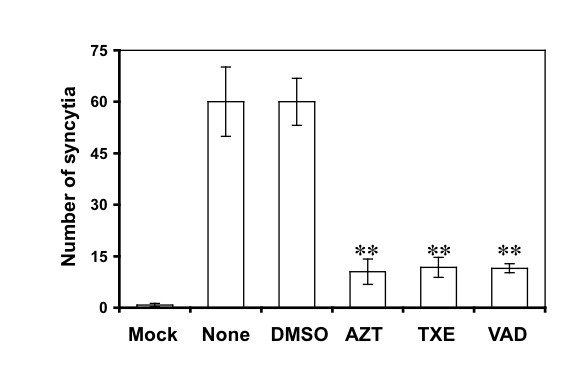
**Effect of the extracts on syncytia formation in HIV-1-infected Jurkat cells**. Jurkat cells were infected with HIV-1 and then exposed to the extracts at 10 μg/ml, 0.1% DMSO, or 5 μM AZT. Syncytia in each of these treatments were counted from 4 random fields from each one of the triplicate samples under a light microscope over the course of 2 weeks infection. The data represented the number of syncytia at day 7 post infection when the maximal number of syncytia was recorded in the infections receiving no treatments (None) or DMSO. Jurkat cells without HIV-1 infection were included as a control (Mock). The data were mean ± SEM of triplicate experiments.

Productive HIV-1 infection of CD4+ T cells in vitro is characterized by formation of multinucleated giant cells, so-called syncytia, which likely results in CD4+ T cell depletion in HIV-1-infected subjects. We next determined effects of these two extracts on syncytia formation. We infected Jurkat cells, treated them with 10 μg/ml TXE or VAD, and monitored the syncytia formation in these cells over a course of 2 weeks. We also included AZT (5 μM) and DMSO (0.1%) as controls. In addition, we also had uninfected and HIV-1-infected Jurkat cells as controls. The number of syncytia reached the highest at day 7 post infection. Compared to untreated and DMSO-treated HIV-1-infected Jurkat cells, TXE, VAD and AZT treatments all showed significant reduction (Figure 3). There were few syncytia in Jurkat cells that received no HIV-1-infection. These results are in agreement with the inhibitory effects of these extracts on HIV-1 replication (Figure 1A).

### No inhibition of HIV-1 RT activity by the extracts

**Figure 4:**
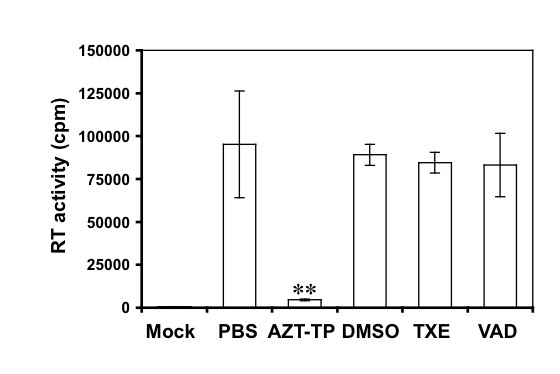
**Direct effects of the extracts on the RT activity**. HIV-1 virions were assayed for their RT activity in the presence of the extracts at 10 μg/ml. AZT-TP (5 μM) was included as a positive control. DMSO (0.1%) and phosphate-buffered saline (PBS) were included as the solvent control for the extracts and AZT, respectively. The RT reaction without any input HIV-1 virions was also included as an assay control. The data were mean ± SEM of triplicate experiments.

HIV-1 is a member of the retrovirus family. The most important feature of these viruses is that replication of these viruses involves conversion of their RNA viral genome to proviral DNA, which is catalyzed by a unique virally encoded enzyme called reverse transcriptase (RT). Therefore, we next determined whether the extracts directly inhibited HIV-1 RT enzymatic activity. To do so, we compared the RT enzymatic activity of HIV-1 virions in the presence and absence of the extracts. We lysed HIV-1 virions to release the RT and performed the RT activity assay in the presence of TXE and VAD (10 μg/ml). We also included the RT inhibitor AZT (5 μM) as a control in these experiments. In addition, we also included PBS and DMSO (0.1%) as the solvent controls for AZT and TXE and VAD, respectively. The RT reaction without any HIV-1 virions was used as an assay blank control. As expected, AZT potently inhibited HIV-1 RT activity. However, inclusion of TXE and VAD in the RT reaction did not show any significance difference in the RT activity from the PBS and DMSO control (Figure 4). These results imply that TXE and VAD-induced inhibition of HIV-1 replication is not due to their effects on the RT activity but on other steps of HIV life cycle.

### Block of HIV-1 entry by these extracts

**Figure 5:**
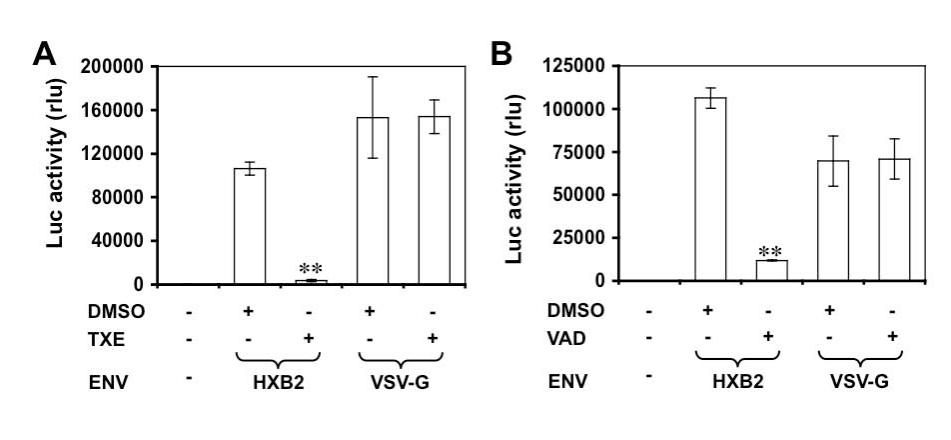
**Effects of the extracts on HIV-1 entry**. U87.CD4.CXCR4 cells were treated with TXE (**A**) or VAD (**B**) at a concentration of 10 μg/ml for 30 min and then infected with HIV-Luc viruses pseudotyped T-tropic HIV-1 HXB2 envelope (HXB2) or without envelope (-) for 2 hr. Forty-eight hours post infection, cells were harvested for the Luc activity assay. HIV-Luc viruses pseudotyped with VSV-G envelope (VSV-G) were included as a control. DMSO (0.1%) was also included as a solvent control for the extracts. The data were mean ± SEM of triplicate experiments.

HIV-1 infection begins with HIV-1 envelope gp120 binding to CD4 and chemokine co-receptors CCR5 (for M-tropic strains) or CXCR4 (for T-tropic strains) on the cell surface of HIV-1 target cells. Thus, we next determined whether the extracts had any effects on HIV-1 entry. To achieve this, we took advantage of the replication-defective single round HIV-Luc reporter system [30]. The replication-defective HIV-Luc reporter system has the HIV-1 *env *gene inactivated and the firefly luciferase (Luc) gene in place of HIV-1 *nef*. Such a design allows *in trans *complementation of any viral envelope proteins including HIV-1 envelope proteins and one single round viral infection to accurately determine HIV-1 entry by the sensitive Luc activity assay. We prepared HIV-Luc reporter viruses pseudotyped with T-tropic HIV-1 HXB2 envelope. To determine effects of these extracts on HIV-1 entry, we pre-incubated U87.CD4.CXCR4 cells with these extracts at 10 μg/ml and then infected these cells with these viruses. We then determined the Luc activity of these cells. We also prepared HIV-Luc reporter viruses pseudotyped with vascular stomatitis virus envelope glycoprotein (VSV-G) or HIV-Luc viruses without any viral envelopes, which were positive and negative controls, respectively, in these experiments. We also included DMSO (0.1%) as the solvent control for these extracts. Compared to the DMSO control, pre-incubation of U87.CD4.CXCR4 with TXE almost completely blocked infection of HIV-Luc pseudotyped with HIV-1 HXB2 envelope but had no effects on that of HIV-Luc pseudotyped with VSV-G (Figure 5A). Similar results were obtained with VAD extract (Figure 5B). These results led us to conclude that TXE and VAD inhibited HIV-1 replication through their block in HIV-1 entry to target cells.

### No effects of the extracts on HIV-1 itself and HIV-1 post-entry

**Figure 6:**
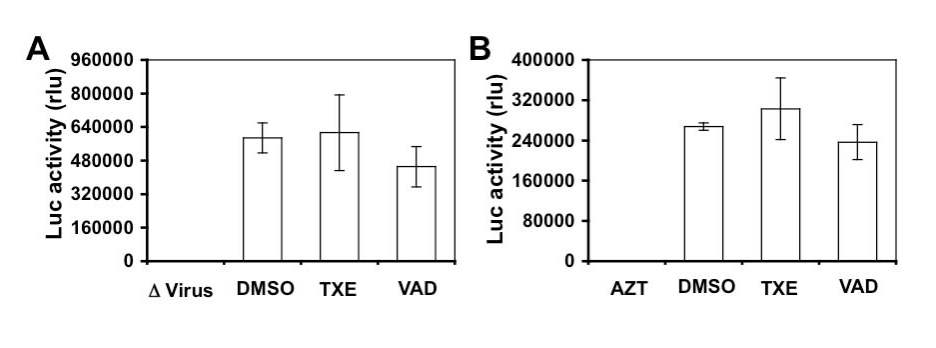
**Effects of the extracts on HIV-1 and HIV-1 gene expression**. **A**. HIV-Luc viruses pseudotyped T-tropic HIV-1 HXB2 envelope (HXB2) were incubated with 10 μg/ml extracts for 2 hr and then used to infect U87.CD4.CXCR4 cells. Cells were harvested 48 hr for the Luc activity assay 48 hr after infection. Infection with heat-inactivated HIV-Luc/HXB2 viruses (Δ Virus) was included as the control. **B**. U87.CD4.CXCR4 cells were infected with HIV-Luc viruses pseudotyped T-tropic HIV-1 HXB2 envelope (HXB2) or without envelope (-) for 2 hr and then removed of the remaining input viruses by repeated washes with fresh medium. Then, the infected cells were cultured for 48 hr in the presence of the extracts (10 μg/ml) and then harvested for the Luc activity assay. DMSO (0.1%) was also included as a solvent control for the extracts, while 0.5 μM AZT was included as a positive control. The data were mean ± SEM of triplicate experiments.

To further ascertain that TXE and VAD inhibited HIV-1 replication at the entry step and to determine whether TXE and VAD directly inactivated HIV-1 and had any effects at other steps of HIV-1 life cycle, we took advantage of the same replication-defective single round HIV-Luc reporter system but with a modified experimental scheme. We first incubated the same amount of HIV-Luc viruses pseudotyped with HXB2 envelope in 10 μg/ml TXE, VAD, or 0.1% DMSO at 37°C for 2 hr. We then recovered the viruses by centrifugation and used them to infect U87.CD4.CXCR4 cells. We cultured the cells for 48 hr before we harvested them for the Luc activity assay. The Luc activity showed no significant difference in the viral infectivity between DMSO treatment control and TXE or VAD treatment, while heat-inactivated viruses had little infectivity (Figure 6A). Next, we first infected U87.CD4.CXCR4 cells with HIV-Luc viruses pseudotyped with HIV-1 HXB2 envelope at 37°C for 2 hr. We then removed the remaining input viruses by repeated washes with fresh medium and cultured these cells for 48 hr in the presence of these extracts (10 μg/ml) or 5 μM AZT. We then harvested the cells for the Luc activity assay. Compared to the AZT control, TXE and VAD treatment did not show any differences in the Luc activity of HIV-infected cells from the DMSO treatment (Figure 6B). Taken together, these results show that the observed inhibition of TRX and VAD on HIV-1 replication is not due to inactivation of HIV-1 by these extracts or the blockage at the post-entry step, further supporting our conclusion that TRE and VAD inhibit HIV-1 replication at the entry step of the viral life cycle.

### Effect of TXE or VAD on entry of primary HIV-1 isolate 89.6 into cells

**Figure 7:**
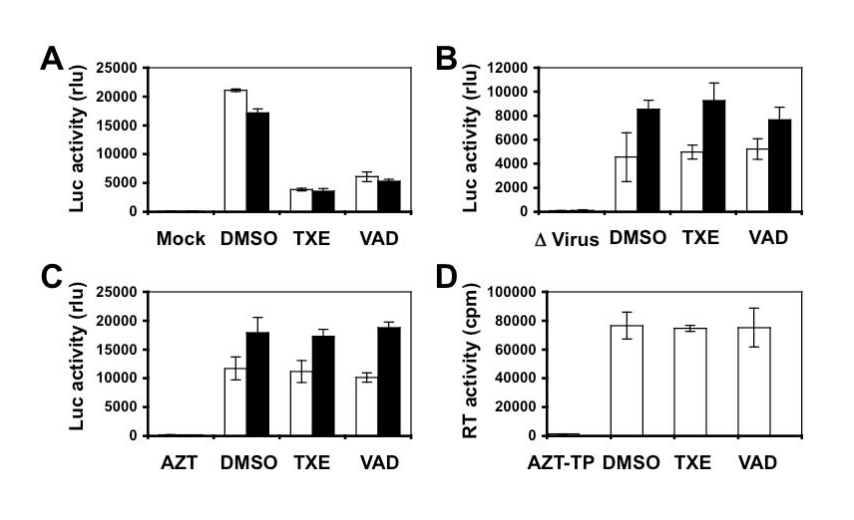
**Effects of the extracts on primary HIV-1 isolate 89.6**. **A**. U87.CD4.CXCR4 and U87.CD4.CCR5 cells were first treated with 10 μg/ml extracts and then infected with HIV-Luc viruses pseudotyped with 89.6 envelope. Infection of HIV-Luc viruses without an envelope was included as the mock infection control. **B**. HIV-Luc viruses pseudotyped 89.6 envelope were first incubated with 10 μg/ml extracts and then used to infect U87.CD4.CXCR4 and U87.CD4.CCR5 cells. Infection with heat-inactivated HIV-Luc/89.6 viruses (Δ Virus) was included as the control. **C**. U87.CD4.CXCR4 and U87.CD4.CCR5 were first infected with HIV-Luc viruses pseudotyped with 89.6 envelope and then treated with 10 μg/ml extracts or 5 μM AZT. **A-C**: open bar for U87.CD4.CXCR4 cells; closed bar for U87.CD4.CCR5 cells. **D**. HIV-Luc viruses pseudotyped with 89.6 envelope were directly treated with 10 μg/ml extracts or 5 μM AZT-TP, the RT activity was determined. The data were mean ± SEM of triplicate experiments.

Next, we extended our experiments to the dual tropic primary HIV-1 isolate 89.6 [31] to investigate whether TXE and VAD were also capable of blocking this virus from entering into its target cells. To this end, we treated U87.CD4.CXCR4 and U87.CD4.CCR5 cells were treated with10 μg/ml of TXE or VAD, and then infected the cells with HIV-Luc viruses pseudotyped with HIV-1 89.6 envelope and performed the Luc activity assay. Compared to the DMSO treatment control, both TXE and VAD treatment showed significant lower Luc activities in both U87.CD4.CXCR4 and U87.CD4.CCR5 cells (Figure 7A), indicating that they blocked HIV-1 89.6 infection. In addition, we also determined effects of the extracts on HIV-1 89.6 itself and at the post-entry step. Similarly to HXB2 viruses, 89.6 viruses did not show any changes in their infectivity when they were exposed to the extracts prior to the infection (Figure 7B) and in their post-entry replication when 89.6 infection occurred prior to the extract treatment (Figure 7C). Furthermore, these extracts did not have any direct inhibitory effects on the RT enzymatic activity of the HIV-1 89.6 viruses (Figure 7D). Taken together, these data demonstrate that TXE and VAD both also possessed the similar activity to block entry of primary HIV-1 isolate 89.6.

### Identification of anti-HIV components of TXE and VAD

**Figure 8:**
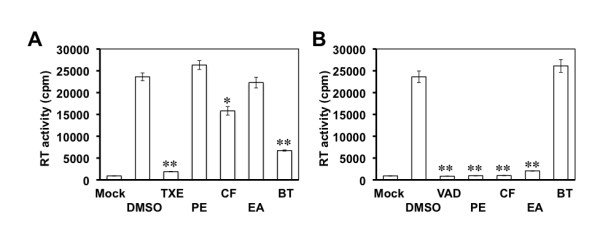
**Anti-HIV components of TXE and VAD extracts**. Jurkat cells were infected with HIV-1 HXB2 and then exposed to 10 μg/ml TXE (**A**), VAD (**B**), or each of its partition subfractions from petroleum ether (PE), chloroform (CF), ethyl acetate (EA) and n-butanol (BT) 24 hr post infection. Fresh extracts or subfraction were added every other day. Meanwhile, culture supernatants were collected for the RT activity assay, and aliquots of cells were stained with trypan blue dye and counted for viable cells. DMSO was the solvent of the extracts and subfractions and included as a vehicle control. The RT data from the supernatants collected at day 9 of the peak viral replication were presented. Extracts and their subfractions showed no apparent cytotoxic effects on the cells. The data were mean ± SEM of triplicate experiments.

As part of further characterization of the anti-HIV activity of these two crude extracts, we next partitioned them into four subfractions using organic solvents of different hydrophobicity and polarity, that is, petroleum ether (PE), chloroform (CF), ethyl acetate (EA) and n-butanol (BT). We then determined the effects of these subfractions on HIV-1 replication. As showed above, TXE treatment inhibited HIV-1 replication in a highly significant fashion (Figure 8A). Compared to the DMSO-treated control, HIV replication was significantly lower in cells treated with CF and BU subfractions, while HIV replication showed little changes in cells treated with its PE and EA subfractions. In contrast, the PE, CF, and EA subfractions of the VAD extract had highly significantly lower HIV-1 replication than the DMSO control, while its BT subfraction displayed no anti-HIV activity (Figure 8B). Similar results were obtained from the single-round infection assay (data not shown). These results suggest that the active anti-HIV components can be further isolated from both TXE and VAD extracts and may differ between these two extracts.

## Discussion

Using a well-established HIV-1 replication system, we screened extracts of traditional Chinese medicinal herbal plants for their anti-HIV activities (Table 1). We showed that extracts from the stem of *Euphorbiaceae*, *Trigonostema xyphophylloides *(TXE) and the stem of *Dipterocarpaceae*, *Vatica astrotricha *(VAD) inhibited HIV-1 replication without apparent effects on cell proliferation and cell survival (Figure 1 and Figure 2). The inhibitory effects of these two extracts were further corroborated by the finding that these extracts prevented HIV-infected cells from forming syncytia (Figure 3). Nevertheless, we did not detect any effects of these extracts on HIV-1 RT enzymatic activity (Figure 4). Instead, we showed that these extracts potently blocked HIV-1 from entering its target cells (Figure 5). Furthermore, we showed that these extracts had little effects on post-entry HIV-1 gene expression (Figure 6). We obtained similar results with the primary isolate, HIV-1 89.6 which displays dual tropism, using both CXCR4 and CCR5 for entry into cells (Figure 7). Taken together, these studies revealed the anti-HIV activities of these two plant extracts and suggest that their anti-HIV activity results from interfering with HIV-1 entry.

All currently FDA-approved anti-HIV drugs are chemically synthesized [32,33]. Development of these drugs involves an extremely long cycle of research, design and optimization, thus these drugs are very expensive. Besides, as many of these drugs are structural analogs of host metabolic components, use of these drugs is often limited by side-effects and non-adherence issues. In contrast, medicines of natural origins such as herbs have a much short development cycle and relatively inexpensive. Importantly, the toxicity of nature-derived medications is rarely an issue. A recent study has shown that more than two third of those who are on anti-HIV medications are also taking alternative therapy, including nearly 25% on those derived from botanicals and Chinese herbs [34,35]. There is clearly a need to further investigate and develop this alternative anti-HIV therapy, as it likely makes the anti-HIV therapy ultimately affordable and available to all HIV/AIDS-affected individuals including those in developing and under-developed countries.

Some efforts have been made to identify natural remedies to combat HIV/AIDS in clinical settings. A number of natural products have been shown to possess anti-HIV-1 activities, including those derived from microorganisms, marine organisms, and plants, and these natural products inhibit HIV-1 replication at various steps of HIV-1 life cycle [36]. Currently, more than 150 natural products have been isolated from marine organisms to show promising anti-HIV-1 activity [37,38]. One of the outstanding examples is cyanovirin-N, an 11 KDa anti-HIV-1 protein that was initially isolated from the cyanobacterium (bluegreen alga) *Nostoc ellipsosporum *[39]. Cyanovirin-N inhibits HIV-1 replication through its vivid binding to HIV-1 gp120 and as a result, inactivates the viruses and blocks the fusion of viruses to the cell membrane [40,41]. The protein is now in Phase II clinical trial to be used as an anti-HIV-1 microbicide.

Traditional Chinese Medicines (TCMs) have a very long history. They have been used to treat various human diseases and regarded as the state cultural treasure by the Chinese government. Development and standardization of TCMs have recently been proposed as the top biomedical research priority for the next 5–10 years in P. R. China. Studies on the anti-HIV activities and mechanisms of TCMs are very limited and are expected to accelerate. Currently, HIV-1-inhibitory TCMs are reported to include *Scutellaria baicalensis Georgi*, Prunella vulgaris, Paeonia Suffruticosa, Rhizoma Polygoni Cuspidati, Radix Notoginseng, Ramulus Visci, and Ajuga Decumbens Thumb [42-47]. Our studies added *Euphorbiaceae*, *Trigonostema xyphophylloides *(TXE) and *Dipterocarpaceae*, *Vatica astrotricha *(VAD) onto this soon-to-be-rapidly-expanding list. Studies are under way to further fractionate these extracts for identification of the active anti-HIV-1 entry constituents in these extracts and for better characterization of their effects on the interaction between HIV-1 gp120 and CD4/chomine receptors CCR5 and CXCR4.

## Conclusion

TXE and VAD extracts possess potent inhibitory activities against HIV-1 replication and entry of both T and M tropic HIV-1 isolates. These results suggest that TXE and VAD are potential biosources for further identification and isolation of active anti-HIV-1 constituents. Identification of these active constituents will help establish the precise mechanisms of this entry inhibition as well as standardize the extracts for potential clinical translation.

## List of abbreviations

TXE: extract from *Euphorbiaceae, Trigonostema xyphophylloides*; VAD: extract from *Dipterocarpaceae, Vatica astrotricha; *TCM: traditional Chinese medicine; HIV-Luc: A HIV-1-based reporter virus containing inactive *nef *and *env *genes and having the luciferase (Luc) gene inserted at the 5' end *nef *gene; 89.6-env: envelope from HIV-1 isolate 89.6; HXB2-env: envelope from HIV-1 isolate HXB2; VSV-G: vascular stomatitis virus envelope glycoprotein; RT: reverse transcriptase; PBS: phosphate-buffered saline; DMSO: dimethyl sulfoxide; AZT: azidothymidine; AZT-TP: azidothymidine triphosphate.

## Competing interests

The authors declare that they have no competing interests.

## Authors' contributions

IWP, CH, GC and JJH designed, organized and coordinated the overall study, and wrote the manuscript. CH and GC coordinated collection and extraction of the plant samples, IWP and JJH designed the HIV-1 experiments, IWP, TW, LG and YL performed the HIV-1 experiments and acquired and analyzed the data. CH, XPS, CC, XMS, BY and GC collected the plant samples and performed extraction. All authors read and approved the final manuscript.

## Pre-publication history

The pre-publication history for this paper can be accessed here:


